# The Association Between Components of Metabolic Syndrome and Abnormal Electrocardiograms in the Saudi Population: A Retrospective Study

**DOI:** 10.7759/cureus.56782

**Published:** 2024-03-23

**Authors:** Sherif M Zaki, Dina S El Karsh, Shahad Yousef, Taif Jamal, Alaa Alsaddah

**Affiliations:** 1 Anatomy, Fakeeh College for Medical Sciences, Jeddah, SAU; 2 Family Medicine, Dr. Soliman Fakeeh Hospital, Jeddah, SAU

**Keywords:** retrospective study, saudi arabia, metabolic syndrome, ecg abnormalities, ecg variables

## Abstract

Review: Saudi Arabia has a high metabolic syndrome (MetS) prevalence. Having MetS increases the risk of cardiovascular disease (CVD), CVD mortality, and myocardial infarction (MI). There is a lack of information regarding MetS and electrocardiogram (ECG) abnormalities in Saudi Arabian populations. Further, it is unclear to what extent MetS components are associated with abnormal ECGs in Saudi populations.

Aim: We investigated whether ECG abnormalities and MetS are associated with Saudi adults. Furthermore, we assessed the relationship between ECG abnormalities and the components of MetS based on the age and gender of the individuals.

Materials and methods: A retrospective study was conducted at Dr Soliman Fakeeh Hospital in Jeddah, Saudi Arabia, on 208 patients with MetS. Participants' clinical and laboratory data were examined. A detailed analysis of the ECG was performed. ECG abnormalities were divided into minor and major abnormalities based on Novacode criteria. In addition to ischemic ECG findings, the ECG showed prolonged PR intervals, prolonged P duration, prolonged QRS duration, and prolonged QTc intervals.

Results: One hundred and thirty-seven participants (65.9%) had elevated fasting blood glucose (FBS), 129 had central obesity (62%), 93 had high blood pressure (BP) (44.7%), 74 had elevated triglycerides (35.6%), and 49 had low high-density lipoprotein (23.6%). An abnormal ECG was found in 86 (41.3%) participants. It consisted of ischemic ECGs, atrioventricular (AV) block (first and second degrees), bundle branch block (right bundle branch block [RBBB], left bundle branch block [LBBB], RBBB with left anterior hemiblock, RBBB with right anterior hemiblock), arrhythmias (premature ventricular contractions [PVCs], premature atrial complexes [PACs], atrial fibrillation [AF], sinus bradycardia, sinus arrhythmia), prolonged QTc, prolonged PR interval, and prolonged QRS duration. There were 29 (13.9%) cases with multiple ECG abnormalities, 57 (27.4%) had one abnormal ECG, 42 (20.2%) had minor abnormal ECGs, and 44 (21.2%) had major abnormal ECGs. Middle-aged and elderly males accounted for the majority of these ECG changes. In the central obesity group, 22 participants (10.6%) had ischemic ECGs, 18 (8.7%) had prolonged QTc, 10 (4.8%) had first-degree AV block, 6 (2.9%) had sinus bradycardia, 7 (3.4%) had RBBB, 4 (1.9%) had LBBB, 3 (1.4%) had PVCs, 2 (1%) had ventricular preexcitation, and one (0.5%) had PACs. An elevated FBS group included 19 participants (9.1%) with an ischemic ECG, 18 (8.7%) with a prolonged QTc, 11 (5.3%) with a first-degree AV block, 9 (4.3%) with sinus bradycardia, 6 (2.9%) with slight ST-T abnormality, 5 (2.4%) with RBBB, and 5 (2.4%) with LBBB. Finally, one (0.5%) of these patients had second-degree AV block, RBBB with left anterior hemiblock, left anterior hemiblock, PVCs, AF, ventricular preexcitation, and sinus arrhythmia for each.

Conclusion:Saudi Arabian populations with MetS were strongly associated with abnormal ECG findings, particularly ischemic ECG findings, AV block (first^ ^and second degrees), and BBB (RBBB, LBBB). Middle-aged and elderly males accounted for the majority of these ECG changes. The most important factors contributing to ECG changes were elevated FBS and central obesity.

## Introduction

Metabolic syndrome (MetS) was first defined by Reaven in 1988 [1]. Between 2002 and 2004, MetS affected 10-23% of the world's population [2,3]. Among the Middle Eastern populations between 2003 and 2014, MetS prevalence was about 25% [4]. The prevalence of MetS in Gulf Cooperation Council countries ranges from 6% to 23.7% [5]. In the United Arab Emirates, MetS is reported to be prevalent in 12% of the population [6]. Saudi Arabia had a MetS incidence rate of 31.6% according to International Diabetes Federation (IDF) guidelines, 39.8% according to Adult Treatment Panel III (ATP III) guidelines, and 40% according to National Cholesterol Education Program (NCEP) guidelines [7,8]. According to a study conducted at King Abdulaziz University Hospital in 2002, 56% of male and 57% of female Saudi diabetics had MetS [9]. 

The NCEP, the IDF, and the World Health Organization (WHO) have all published definitions of MetS [10-12]. In recent years, the NCEP definition has become the most widely used description of MetS [10,13]. NCEP defines MetS as the presence of three or more of the following five clinical findings: central obesity (waist circumference >102 cm for men, 88 cm for women); elevated triglycerides (TG ≥150 mg/dL); decreased high-density lipoprotein (men HDL <40 mg/dL; women HDL <50 mg/dL); systemic hypertension (≥130/≥85 mm Hg); and elevated fasting glucose (≥110 mg/dL) [2,14]. In 2004, the NCEP definition was revised (rNCEP) to lower the threshold for fasting blood glucose (FBS) levels at 100 mg/dL, while central obesity for both men and women was lowered from >102 cm and >88 cm to greater than or equal to these values [14]. As part of the rNCEP definition, patients who are being treated for hypertension, hyperglycemia, or dyslipidemia are also included [14].

Having MetS increases the risk of cardiovascular disease (CVD), CVD mortality, myocardial infarction (MI), and stroke twofold [1]. Several studies have consistently demonstrated that MetS is a significant predictor of coronary artery disease (CAD) [15,16]. In Saudi Arabia, 6.4% of men and 4.4% of women are reported to have CAD [17]. Overall, 5.5% of Saudi Arabians suffer from CAD [18].

There is a lack of information regarding MetS and ECG abnormalities in Saudi Arabian populations. Further, it is unclear to what extent MetS components are associated with abnormal ECGs in Saudi populations. Therefore, we investigated whether ECG abnormalities and MetS are associated with Saudi adults. Furthermore, we assessed the relationship between ECG abnormalities and the components of the MetS based on the age and gender of the individuals. 

## Materials and methods

The retrospective study analyzed 208 patients with MetS who regularly attend the family medicine clinic at Dr. Soliman Fakeeh Hospital in Jeddah, Saudi Arabia. The study took place between July 2023 and January 2024. Consent was obtained or waived by all participants in this study. The Institutional Review Board at Fakeeh College for Medical Sciences issued approval 523/IRB/2023. Patients under 18 years of age and participants who did not want to participate in the study were excluded.

Participants must meet the rNCEP definitions for MetS [14]. The modified rNCEP definitions typically used body mass index (BMI >30 kg/m^2^) instead of waist circumference to define central obesity [14]. The patients had to meet three or more of the following criteria: 1) central obesity (BMI >30 kg/m^2^); 2) elevated TG (≥150 mg/dL); 3) decreased HDL (<40 mg/dL for men; <50 mg/dL for women); 4) FBS (≥100 mg/dL), or patients known to have diabetes or on diabetes treatment; 5) high systolic/diastolic blood pressure (SBP ≥ 130 mmHg and/or DBP ≥ 85 mmHg), or on hypertension treatment [14].

Using medical records, we collected socio-demographic information, including age, sex, height, weight, BMI, exercise, and smoking history. Moreover, family histories of hypertension, diabetes, and CVD were obtained. Patients' records were also reviewed for histories of angina and its medication (nitroglycerin), MI, and cerebrovascular (CV) strokes. 

Hemoglobin, FBS, hemoglobin A1C, total cholesterol (mmol/L), TG (mmol/L), low-density lipoprotein (mmol/L), and HDL (mmol/L) levels were determined from the latest blood test results. The results of non-interventional procedures such as blood pressure (BP) and echocardiogram (echo) as well as interventional procedures such as coronary bypass surgery and cardiac catheterization were also obtained from the patient's records.

Standard 12-lead ECGs were recorded with the patient supine and according to standardized procedures. We visually examined all ECGs for improper quality, missing leads, and technical errors. ECG device provides information about the duration, amplitude, and axis of ECG waves. Heart rate (bpm), P amplitude II (mV), PR interval (mS), QRS duration (mS), R amplitude V5 (mV), S amplitude V1 (mV), QT interval (mS), QTc interval (mS), P axis (°), and QRS axis (°) were also collected.

As well, prolonged PR intervals were defined as PR intervals ≥200 ms, prolonged P duration as P duration ≥120 ms, and prolonged QRS duration as QRS duration ≥100 ms. We used clinically accepted cutoffs for prolonged QTc intervals (Man >450 ms, woman >470 ms). Furthermore, the Sokolow-Lyon index is calculated as the sum of S amplitudes V1 and R amplitudes V5/6 [19].

ECG abnormalities were divided into minor and major abnormalities based on Novacode criteria [20]. Minor ECG abnormalities included any of the following: 1) first- and second-degree atrioventricular block (AV block); 2) borderline prolonged ventricular excitation; 3) prolonged ventricular repolarization; 4) isolated minor Q and ST-T abnormalities; 5) left ventricular hypertrophy without ST-T abnormalities; 6) left atrial enlargement; 7) frequent atrial or ventricular premature beats (premature atrial complexes [PACs], premature ventricular contractions [PVCs]); and 8) fascicular blocks. Major ECG abnormalities included any of the following: 1) atrial fibrillation (AF); 2) high-degree AV dissociation; 3) left bundle branch block (LBBB); 4) right bundle branch block (RBBB); 5) indeterminate conduction delay; 6) Q-wave MI; 7) isolated ischemic abnormalities; 8) left ventricular hypertrophy with ST-T abnormalities; and 9) miscellaneous arrhythmias (e.g., supraventricular tachycardia, ventricular preexcitation, ventricular tachycardia).

Ischemic ECG findings (MI or ischemia) were defined as the presence of Q/Qs patterns, significant or borderline ST segment depression, deep or moderate T wave inversion, or evidence of complete LBBB [21].

Statistical analysis

Statistical Package for Social Sciences (SPSS), version 21 (IBM Corp, Armonk, NY), was used to analyze the data. Data was summarized by frequency and percentage for qualitative data and by means and standard deviations for quantitative data. An independent-sample t-test was conducted to determine the statistical significance of the difference in continuous variables according to gender and age groups. The chi-square (χ^2^) test was used to compare the prevalence rates of MetS components and ECG abnormalities by gender and age. The chi-square test was also used to study the association between participants' ECG abnormalities and MetS components. As a last step, we calculated the odds ratio (OR) of the ECG abnormalities, prolonged PR, prolonged QRS, prolonged QTc, and ischemic ECG changes.

## Results

There were 208 participants in this study with MetS, 110 males and 98 females. The average age of all participants was 59 ± 10 years, 58 ± 12 years for the male, and 59 ± 8 years for the female. Table 1 lists the clinical and laboratory characteristics of the study participants.

**Table 1:** Participant characteristics based on clinical and laboratory examinations *p < 0.05. LDL, low-density lipoprotein; HDL, high-density lipoprotein; BMI, body mass index; systolic BP, systolic blood pressure; diastolic BP, diastolic blood pressure; Hb, hemoglobin; HbA1c, hemoglobin A1c.

Characteristics	Male	Female	Total	p-Value
	Mean ± SD	Mean ± SD	Mean ± SD	
Age (years)	58 ± 12	59 ±8	59 ± 10	0.001*
Height (cm)	170 ± 7	156 ± 6	163 ± 10	0.184
Weight (kg)	91 ± 18	83 ± 16	87 ± 18	0.200
Systolic BP (mmHg)	130 ± 14	124 ± 17	127 ± 16	0.239
Diastolic BP (mmHg)	78 ± 8	75 ± 7	76 ± 8	0.690
BMI (kg/m^2^)	31 ± 5	34 ± 6	33 ± 6	0.222
Fasting plasma glucose (mg/dL)	130 ± 40	128 ± 41	129 ± 41	0.452
Triglyceride (mmol/L)	145 ± 72	129 ± 48	137 ± 62	0.206
Total cholesterol (mmol/L)	174 ± 51	182 ± 45	177 ± 49	0.000*
LDL (mmol/L)	110 ± 53	115 ± 40	113 ± 47	0.043*
HDL (mmol/L)	45 ± 9	51 ± 13	47 ± 11	0.230
HBA1c	7 ± 2	7 ± 1	7 ± 1	0.016*
Hb level	15 ± 2	13 ± 2	14 ± 2	0.290

Based on Table 2, 137 participants (65.9%) had elevated FBS, 129 (62.0%) had central obesity, 93 (44.7%) had high BP, 74 (35.6%) had elevated TG, and 49 (23.6%) had low HDL. Participants' elevated TG and FBS did not differ significantly based on their demographics. There was a significant difference between participants by age and sex in terms of high BP and central obesity. A significant difference was found between males (36 (17.31%)) and females (13 (6.3%)) when it came to low HDL levels, but no significant differences were found by age group.

**Table 2:** The frequency of metabolic syndrome components among participants *p < 0.05 by χ^2^ test. TG, triglyceride; HDL, high-density lipoprotein; FBS, fasting plasma glucose; BP, blood pressure; CAD, coronary artery disease.

Characteristics	Elevated TG		Low HDL		Elevated FBS		High BP		Central obesity		Total	
	Number	%	Number	%	Number	%	Number	%	Number	%	Number	%
Age group												
Young adult	3	1.4%	5	2.4%	5	2.4%	4	1.9%*	10	4.8%*	10	4.8%
Middle-aged adult	40	19.2%	23	11.1%	65	31.3%	34	16.3%*	66	31.7%*	99	47.6%
Old adult	31	14.9%	21	10.1%	67	32.2%	55	26.4%*	53	25.5%*	99	47.6%
Sex												
Male	41	19.7%	36	17.3%*	67	32.2%	59	28.4%*	59	28.4%*	110	52.9%
Female	33	15.9%	13	6.3%*	70	33.7%	34	16.3%*	70	33.7%*	98	47.1%
Smoking												
Non-smoking	38	18.3%	18	8.7%*	83	39.9%	48	23.1%*	85	40.9%*	123	59.1%
Smoking	36	17.3%	31	14.9%*	54	26.0%	45	21.6%*	44	21.2%*	85	40.9%
Exercise												
Non-exercising	71	34.1%	48	23.1%	132	63.5%	89	42.8%	122	58.7%	198	95.2%
Exercising	3	1.4%	1	0.5%	5	2.4%	4	1.9%	7	3.4%	10	4.8%
Family history												
Positive family history of hypertension	71	34.1%	46	22.1%	130	62.5%	83	39.9%*	123	59.1%	195	93.8%
Positive family history of diabetes	72	34.6%	46	22.1%	134	64.4%*	88	42.3%	123	59.1%	199	95.7%
Positive family history of CAD	32	15.4%	28	13.5%*	59	28.4%	46	22.1%*	53	25.5%	86	41.3%
Total	74	35.6%	49	23.6%	137	65.9%	93	44.7%	129	62.0%	208	100.0%

There were 85 smokers (40.9%) among the participants. The smoking group had 31 (14.9%) low HDL, 45 (21.6%) high BP, 44 (21.2%) central obesity, 36 (17.3%) elevated TGs, and 54 (26.0%) elevated FBS (Table 2).

A total of 198 (95.2%) participants did not exercise. A total of 48 (23.1%) of the non-exercising group had low HDL, 89 (42.8%) had high BP, 122 (58.7%) had central obesity, 71 (34.1%) had elevated TG, and 132 (63.5%) had elevated FBS (Table 2).

There were 199 (95.7%) with a positive family history of diabetes, 195 (93.8%) with a positive family history of hypertension, and 86 (41.3%) with a positive family history of CAD (Table 2). 

CVD prevalence is listed by age and sex in Table 3. Seven patients (3.4%) had angina, while 10 (4.8%) developed MI. A total of 86 (41.3%) participants had abnormal ECGs, and 61 (29.5%) had abnormal echo. Thirty-one (14.9%) patients had cardiac catheterizations, with a significant difference between males and females. Males make up all MI participants. The majority of patients (5.3%) who needed coronary bypass surgery were males. Moreover, 39 (18.8%) participants had peripheral vascular disease and 8 (3.8%) had CV strokes (Table 3).

**Table 3:** Cardiovascular disease prevalence by sex and age *p < 0.05 by χ^2^ test. CVD, cardiovascular disease.

CVD	Male		Female		Young adult		Middle-aged adult		Old adult		Total	
	Number	%	Number	%	Number	%	Number	%	Number	%	Number	%
Angina (nitroglycerine intake)	6	2.9%	1	0.5%	0	0.0%	2	1.0%	5	2.4%	7	3.4%
Myocardial infarction	10	4.8%*	0	0.0%*	1	0.5%	4	1.9%	5	2.4%	10	4.8%
ECG changes	51	24.5%	35	16.8%	3	1.5%	37	17.7%	46	22.1%	86	41.3%
Echo abnormalities	32	15.4%	29	13.9%	3	1.4%	33	15.9%	25	12.0%	61	29.3%
Cardiac catheterization	28	13.5%*	3	1.4%*	1	0.5%	11	5.3%	19	9.1%	31	14.9%
Coronary bypass surgery	11	5.3%*	1	0.5%*	0	0.0%	3	1.4%	9	4.3%	12	5.8%
Peripheral vascular disease	24	11.5%	15	7.2%	0	0.0%	5	2.4%	34	16.3%	39	18.8%
Cerebrovascular stroke	3	1.4%	5	2.4%	0	0.0%	3	1.4%	5	2.4%	8	3.8%
Total	110	52.9%	98	47.1%	10	4.8%	99	47.6%	99	47.6%	208	100.0%

Table 4 lists the ECG variables of the study participants. QTc and QT differ significantly based on sex.

**Table 4:** ECG variables of the study participants *p < 0.05 by t-test. mS, millisecond; mV, millivolt.

ECG variables	Male	Female	Total	p-Value
	Mean ± SD	Mean ± SD	Mean ± SD	
Heart rate	71.41 ± 11.20	72.48 ± 11.03	71.90 ± 11.10	0.530
P duration (ms)	111.80 ± 17.64	109.38 ± 23.86	110.67 ± 20.75	0.451
PR interval (ms)	173.87 ± 33.07	164.71 ± 35.86	169.64 ± 34.59	0.084
QRS duration (ms)	101.03 ± 21.39	103.08 ± 33.54	101.98 ± 27.64	0.632
QT (ms)	373.94 ± 27.12	385.05 ± 41.50	379.14 ± 34.93	0.039*
QTc (ms)	406.13 ± 20.92	425.79 ± 29.70	415.26 ± 27.14	0.000*
P axis	51.54 ± 21.31	49.93 ± 20.42	50.80 ± 20.86	0.625
QRS axis	29.44 ± 44.03	29.30 ± 35.06	29.38 ± 40.03	0.983
T axis	42.30 ± 31.35	46.68 ± 37.94	44.36 ± 34.56	0.422
RV5+SV1 amp (mV)	1.79 ± 0.60	1.69 ± 0.60	1.75 ± 0.60	0.334

Twenty-nine (13.9%) cases had multiple ECG abnormalities, 57 (27.4%) had one ECG abnormality, 42 (20.2%) had minor ECG abnormalities, and 44 (21.2%) had major ECG abnormalities (Table 5).

**Table 5:** Study participants' ECG abnormalities according to their age and gender *p < 0.05 by χ^2^ test. AV, atrioventricular; BBB, bundle branch block; RBBB, right bundle branch block; LBBB, left bundle branch block; PVCs, premature ventricular contractions; PACs, premature atrial complexes; AF, atrial fibrillation.

ECG findings	Male		Female		Young adult		Middle-aged adult		Old adult		Total	
	Number	%	Number	%	Number	%	Number	%	Number	%	Number	%
Normal ECG	59	28.4%	63	30.3%	7	3.4%	62	29.8%	53	25.5%	122	58.7%
Multiple ECG abnormalities	19	9.1%	10	4.8%	1	0.5%	8	3.8%	20	9.6%	29	13.9%
One ECG change	32	15.4%	25	12.0%	2	1.0%	29	13.9%	26	12.5%	57	27.4%
Minor ECG abnormalities	23	11.1%	19	9.1%	0	0.0%	23	11.1%	19	9.1%	42	20.2%
Major ECG abnormalities	28	13.5%	16	7.7%	3	1.4%	14	6.7%	27	13.0%	44	21.2%
Ischemic ECG	23	11.1%	11	5.3%	3	1.4%	11	5.3%	20	9.6%	34	16.3%
AV block	11	5.3%	6	2.9%	0	0.0%*	11	5.3%*	6	2.9%*	17	8.2%
First-degree AV block	9	4.3%	6	2.9%	0	0.0%	9	4.3%	6	2.9%	15	7.2%
Second-degree AV block	2	1.0%	0	0.0%	0	0.0%	2	1.0%	0	0.0%	2	1.0%
BBB	8	3.8%	8	3.8%	0	0.0%*	3	1.4%*	13	6.3%*	16	7.7%
RBBB	3	1.4%	4	1.9%	0	0.0%	1	0.5%	6	2.9%	7	3.4%
LBBB	2	1.0%	4	1.9%	0	0.0%	2	1.0%	4	1.9%	6	2.9%
RBBB with left anterior hemiblock	1	0.5%	0	0.0%	0	0.0%	0	0.0%	1	0.5%	1	0.5%
Left anterior hemiblock	2	1.0%	0	0.0%	0	0.0%	0	0.0%	2	1.0%	2	1.0%
PVCs	2	1.0%	3	1.4%	0	0.0%	2	1.0%	3	1.4%	5	2.4%
PACs	1	0.5%	1	0.5%	0	0.0%	1	0.5%	1	0.5%	2	1.0%
AF	2	1.0%	0	0.0%	0	0.0%	0	0.0%	2	1.0%	2	1.0%
Ventricular preexcitation	1	0.5%	1	0.5%	0	0.0%	1	0.5%	1	0.5%	2	1.0%
Sinus bradycardia	8	3.8%	4	1.9%	1	0.5%	5	2.4%	6	2.9%	12	5.8%
Sinus arrhythmia	1	0.5%	0	0.0%	0	0.0%	0	0.0%	1	0.5%	1	0.5%
Prolonged QTc	3	1.4%*	20	9.6%*	0	0.0%	12	5.8%	11	5.3%	23	11.1%
Slight ST-T abnormality	3	1.4%	5	2.4%	0	0.0%	6	2.9%	2	1.0%	8	3.8%
Repolarization disturbance	2	1.0%	0	0.0%	0	0.0%	0	0.0%	2	1.0%	2	1.0%

Twenty-three (11.1%) of the 34 (16.3%) patients with ischemic ECG changes were men, and 11 (5.3%) were women. Middle-aged and elderly males accounted for the majority of these ECG changes (Table 5).

There were 17 (8.2%) cases of AV block, 11 (5.3%) were males and 6 (2.9%) were females. Middle-aged adults and older adults accounted for all of these cases. Two participants (1%) had second-degree AV block, while 15 (7.2%) had first-degree AV block. BBB was observed in 16 (7.7%) cases, which were equally distributed between men and women. All of these cases involved middle-aged and older adults. Seven (3.4%) BBB cases had RBBB, six (2.9%) had LBBB, one (0.5%) had RBBB with left anterior hemiblock, and two (1.0%) had right anterior hemiblock (Table 5).

There were also other arrhythmias observed in the study, including PVCs, PACs, and AF. PACs, AF, and ventricular preexcitation were observed in 2 (1.0%) cases each, whereas PVCs were observed in 5 (2.4%). There were 12 cases of sinus bradycardia (5.8%) and one case of sinus arrhythmia (0.5%) (Table 5).

There were 23 (11.1%) prolonged QTc cases among middle-aged and older adults. Three male patients (1.4%) and 20 female patients (9.6%) had prolonged QTc, respectively. A right-axis deviation was observed in 6 (2.9%) participants and a left-axis deviation was observed in 8 (3.8%) participants (Table 5).

ECG findings, such as slight ST-T abnormalities and repolarization disturbances were also noted in eight (3.8%) and two cases (1.0%), respectively. The middle-aged and older adults had all these minor findings (Table 5). 

**Figure 1:**
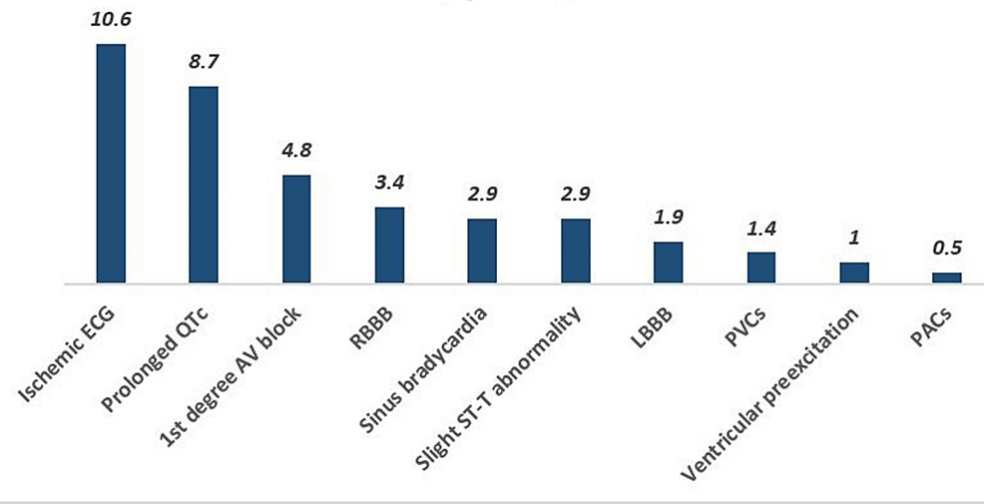
The frequency of abnormal ECGs among central obesity participants RBBB, right bundle branch block; LBBB, left bundle branch block; PVCs, premature ventricular contractions; PACs, premature atrial complexes.

Table 6 shows the frequency of ECG abnormalities associated with MetS components. In the central obesity group, 22 participants (10.6%) had ischemic ECGs, 18 (8.7%) had prolonged QTc, 10 (4.8%) had AV blocks of the first degree, 6 (2.9%) had sinus bradycardia, 7 (3.4%) had RBBB, 4 (1.9%) had LBBB, 3 (1.4%) had PVCs, 2 (1%) had ventricular preexcitation, and one (0.5%) had PACs (Table 6, Figure 1). 

**Table 6:** Participants' ECG abnormalities with metabolic syndrome components *p < 0.05 by χ^2^ test. AV, atrioventricular; BBB, bundle branch block; RBBB, right bundle branch block; LBBB, left bundle branch block; PVCs, premature ventricular contractions; PACs, premature atrial complexes; AF, atrial fibrillation; LA, left atrium; LV, left ventricle; RA, right atrium; TG, triglyceride; HDL, high-density lipoprotein; FBS, fasting plasma glucose; BP, blood pressure.

ECG findings	Elevated TG		Low HDL		Elevated FBS		High BP		Central obesity		Total	
	Number	%	Number	%	Number	%	Number	%	Number	%	Number	%
Ischemic ECG	18	8.7%*	11	5.3%	19	9.1%	17	8.2%	22	10.6%	34	16.3%
AV block	9	4.4%	4	1.9%	12	5.8%	5	2.4%	10	4.8%	17	8.2%
First-degree AV block	7	3.4%	3	1.4%	11	5.3%	4	1.9%	10	4.8%	15	7.2%
Second-degree AV block	2	1.0%	1	0.5%	1	0.5%	1	0.5%	0	0.0%	2	1.0%
BBB	8	3.9%	3	1.4%	12	5.8%	8	3.9%	11	5.3%	16	7.7%
RBBB	2	1.0%	0	0.0%	5	2.4%	3	1.4%	7	3.4%	7	3.4%
LBBB	4	1.9%	3	1.4%	5	2.4%	2	1.0%	4	1.9%	6	2.9%
RBBB with left anterior hemiblock	1	0.5%	0	0.0%	1	0.5%	1	0.5%	0	0.0%	1	0.5%
Left anterior hemiblock	1	0.5%	0	0.0%	1	0.5%	2	1.0%	0	0.0%	2	1.0%
PVCs	3	1.4%	1	0.5%	1	0.5%	4	1.9%	3	1.4%	5	2.4%
PACs	1	0.5%	1	0.5%	0	0.0%	1	0.5%	1	0.5%	2	1.0%
AF	1	0.5%	0	0.0%	1	0.5%	2	1.0%	0	0.0%	2	1.0%
Ventricular preexcitation	0	0.0%	0	0.0%	1	0.5%	0	0.0%	2	1.0%	2	1.0%
Sinus bradycardia	6	2.9%	3	1.4%	9	4.3%	5	2.4%	6	2.9%	12	5.8%
Sinus arrhythmia	0	0.0%	0	0.0%	1	0.5%	0	0.0%	0	0.0%	1	0.5%
Prolonged QTc	11	5.3%	6	2.9%	18	8.7%	11	5.3%	18	8.7%	23	11.1%
Slight ST-T abnormality	6	2.9%	3	1.4%	6	2.9%	3	1.4%	6	2.9%	8	3.8%
Dilated LA	1	0.5%	1	0.5%	3	1.4%	1	0.5%	1	0.5%	3	1.4%
LV hypertrophy	1	0.5%	1	0.5%	1	0.5%	0	0.0%	1	0.5%	1	0.5%
Mild concentric LVH + dilated LA	0	0.0%	0	0.0%	0	0.0%	1	0.5%	0	0.0%	1	0.5%
Dilated RA	1	0.5%	1	0.5%	1	0.5%	1	0.5%	0	0.0%	1	0.5%
Total	74	35.6%	49	23.6%	137	65.9%	93	44.7%	129	62.0%	208	100.0%

**Figure 2:**
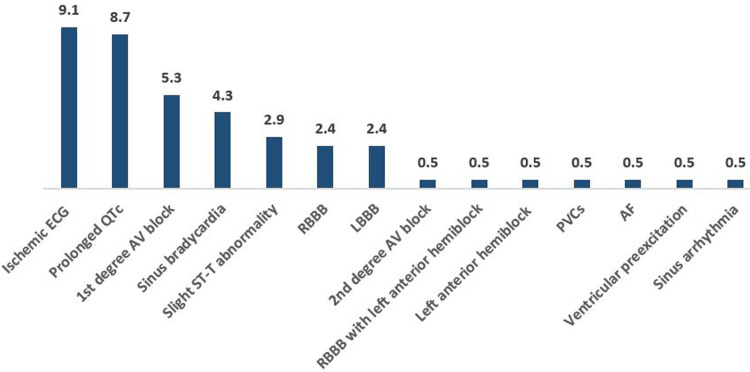
The frequency of abnormal ECGs among elevated FBS participants RBBB, right bundle branch block; LBBB, left bundle branch block; PVCs, premature ventricular contractions; AF, atrial fibrillation; FBS, fasting plasma glucose.

An elevated FBS group included 19 participants (9.1%) with an ischemic ECG, 18 (8.7%) with a prolonged QTc, 11 (5.3%) with first-degree AV block, 9 (4.3%) with sinus bradycardia, 6 (2.9%) with slight ST-T abnormality, 5 (2.4%) with RBBB, and 5 (2.4%) with LBBB. Finally, one (0.5%) of these patients had second-degree AV block, RBBB with left anterior hemiblock, left anterior hemiblock, PVCs, AF, ventricular preexcitation, and sinus arrhythmia for each (Table 6, Figure 2). 

**Figure 3:**
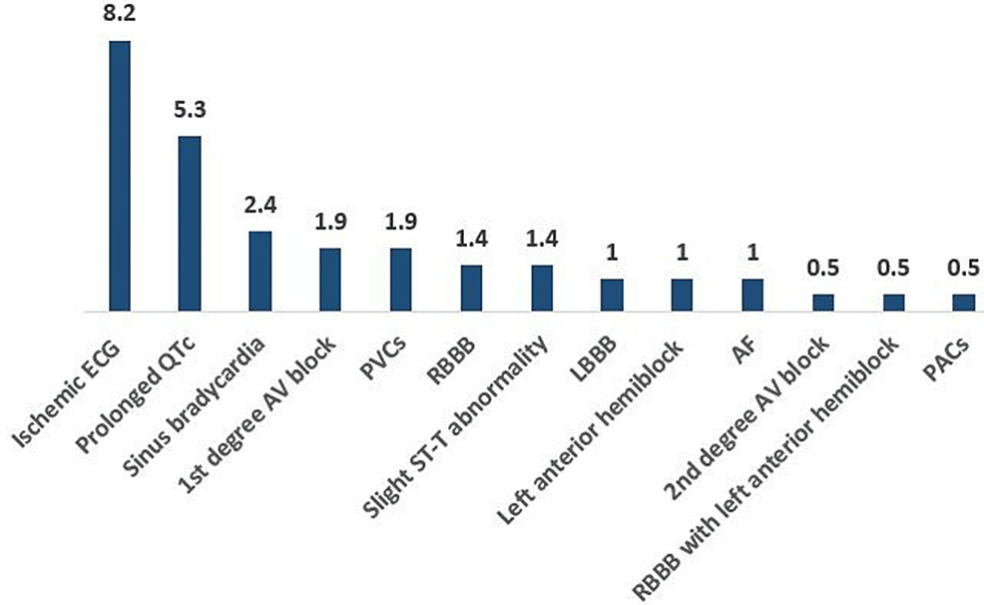
The frequency of abnormal ECGs among high-blood pressure participants RBBB, right bundle branch block; LBBB, left bundle branch block; PVCs, premature ventricular contractions; AF, atrial fibrillation; PACs, premature atrial complexes.

In high-BP patients, 17 (8.2%) participants had ischemic ECGs, 11 (5.3%) had prolonged QTc, 5 (2.4%) had sinus bradycardias, 4 (1.9%) had first-degree AV block, 4 (1.9%) with PVC, 4 (1.9 %) with slight ST-T abnormalities, and 3 (1.4%) with RBBB. Furthermore, 2 (1%) participants in this group had LBBB, left anterior hemiblock, and AF. Finally, one patient (0.5%) had second-degree AV block, RBBB with left anterior hemiblock, and PACs for each (Table 6, Figure 3). 

**Figure 4:**
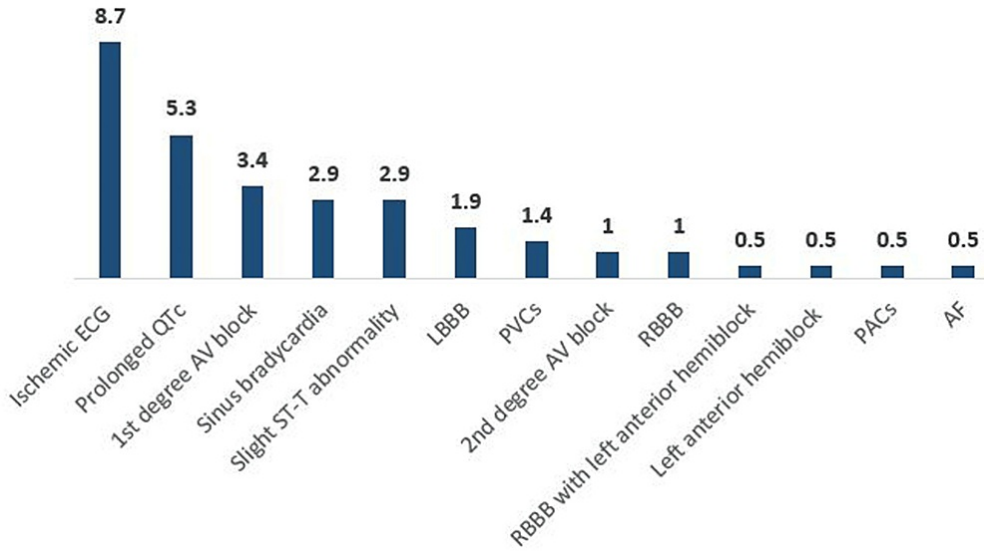
The frequency of abnormal ECGs among elevated TG participants RBBB, right bundle branch block; LBBB, left bundle branch block; PVCs, premature ventricular contractions; AF, atrial fibrillation; PACs, premature atrial complexes; TG, triglyceride.

In elevated TG patients, 18 (8.7%) had ischemic ECGs, 11 (5%) had prolonged QTc, 7 (3.4%) had first-degree AV block, 6 (2.9%) had sinus bradycardia, 6 (2.9%) had slight ST-T abnormalities, 4 (1.9%) had LBBB, 3 (1.4%) had PVCs, 2 (1%) had second-degree AV block, and 2 (1%) had RBBB. Finally, one patient (0.5%) had RBBB, left anterior hemiblock, left anterior hemiblock, AFs, and PACs for each (Table 6, Figure 4). 

**Figure 5:**
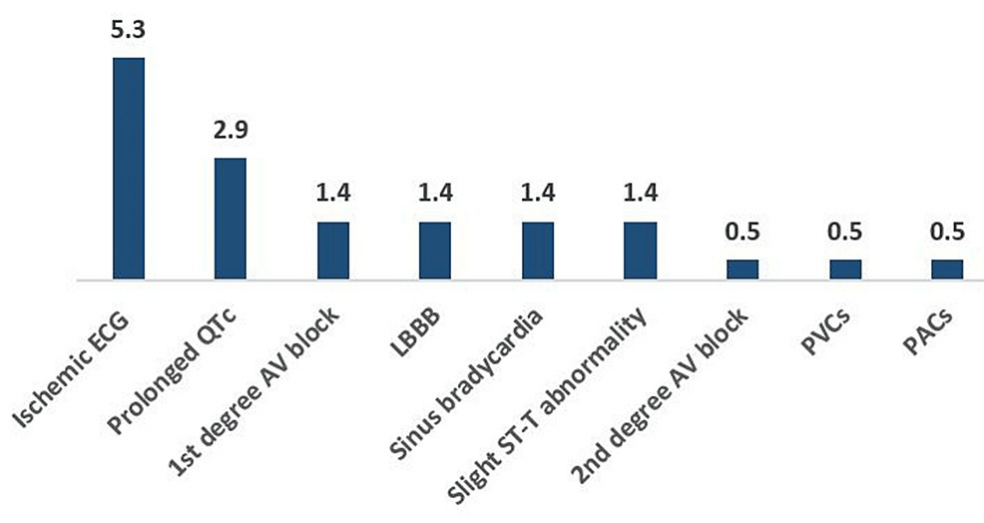
The frequency of abnormal ECGs among low-HDL participants LBBB, left bundle branch block; PVCs, premature ventricular contractions; PACs, premature atrial complexes; HDL, high-density lipoprotein.

A total of 11 (5.3%) patients with low HDL had ischemic ECGs and 6 (2.9%) had prolonged QTc. Three patients (1.4%) had first-degree AV block, LBBB, sinus bradycardia, and slight ST-T abnormality for each. One patient (0.5%) had second-degree AV block, PVCs, and PACs for each (Table 6, Figure 5). 

Table 7 shows the OR of MetS components and ECG abnormalities. 

**Table 7:** Metabolic syndrome components and ECG abnormalities: odds ratios OR, odds ratio; CI, confidence interval; TG, triglyceride; HDL, high-density lipoprotein; FBS, fasting plasma glucose; BP, blood pressure.

ECG findings	Elevated TG			Low HDL			Elevated FBS			High BP			Central obesity		
	OR	CI lower	CI upper	OR	CI lower	CI upper	OR	CI lower	CI upper	OR	CI lower	CI upper	OR	CI lower	CI upper
ECG abnormalities	1.044	0.591	1.845	0.588	0.304	1.136	1.100	0.619	1.956	0.831	0.480	1.438	1.372	0.780	2.414
Prolonged PR (≥200 ms)	2.400	0.983	5.861	0.696	0.224	2.165	1.433	0.535	3.838	0.328	0.116	0.925	0.872	0.354	2.144
Prolonged QRS (≥100 ms)	1.307	0.704	2.426	0.861	0.419	1.768	0.855	0.457	1.600	1.117	0.612	2.040	1.636	0.861	3.109
Prolonged QTc	1.775	0.742	4.249	1.166	0.433	3.141	1.997	0.709	5.623	1.151	0.483	2.743	2.400	0.854	6.746
Ischemic ECG changes	0.422	0.200	0.888	1.712	0.766	3.823	0.601	0.285	1.270	1.289	0.618	2.692	1.148	0.533	2.471

## Discussion

MetS is characterized by elevated FBS in the majority of our study participants (65.9%), followed by central obesity (62%), elevated BP (44.7%), elevated TG (35.6%), and low HDL (23.6%). Various researchers reported different prevalences; high BP, 80.7%; central obesity, 67%; low HDL, 68.7%; and hyperglycemia, 30% [22]. According to some studies, Saudis' MetS was characterized by low HDL levels followed by central obesity [8]. Differences in lifestyles and socioeconomic status may account for these differences.

Among our participants, elevated FBS and central obesity were the most common MetS components. There is considerable evidence that insulin resistance and central obesity are key components of MetS, which contribute to glucose intolerance and dysglycemia [1]. As insulin resistance progresses, peripheral vasoconstriction and sodium retention are triggered, along with hyperinsulinemia and hyperglycemia [1]. Furthermore, central obesity causes systemic hypertension and dyslipidemia independently and via insulin resistance [1].

Aside from a positive family history of diabetes and hypertension, the reasons for high frequencies of the MetS components included aging, being male, smoking, and not exercising. There were 95.2% of participants who were middle-aged or older, and 52.9% of them were males. 95.2% of the study participants did not exercise and 40.9% smoked as part of an unhealthy lifestyle. Some studies have linked smoking and low levels of activity to a greater prevalence of MetS [23,24]. It appears unhealthy lifestyles run in families, as 95.7% of our participants had a positive family history of diabetes, and 93.8% had hypertension.

MetS has been reported to be a significant predictor of CAD, CVD, and MI risk by many authors [1,15,16]. Our patients developed angina in 3.4% of cases and MI in 4.8%, and had coronary bypass surgery in 5.8% and cardiac catheterizations in 14.9%.

A CV stroke occurred in 3.8% of our study participants. Prothrombotic and proinflammatory states caused by lipid imbalances (low HDL cholesterol, hypertriglyceridemia) may explain such strokes. Stroke may also be associated with left ventricular hypertrophy, as previously described by some authors [22]. Finally, stroke may be caused by obesity because obesity prolongs QT and QTc intervals and reduces heart rate variability; these factors may contribute to arrhythmia and cardio-embolic stroke [22].

Among our participants, 41.3% had abnormal ECGs. The abnormal ECGs included ischemic ECG, AV block (first, second degrees), BBB (RBBB, LBBB, RBBB with left anterior hemiblock, RBBB with right anterior hemiblock), arrhythmias (PVCs, PACs, AF, sinus bradycardia, sinus arrhythmia), prolonged QTc, prolonged PR interval, and prolonged QRS duration. MetS may affect ECG variables directly, indirectly, or in a complex manner [25]. ECG abnormalities are prevalent due to high levels of hypertension and dyslipidemia, the most important factors contributing to ECG changes [1].

A total of 24.5% of males and 16.8% of females in our study had abnormal ECG findings. Middle-aged and elderly males accounted for the majority of the abnormal ECG findings. According to Ebong et al., 65.1% of men and 50% of women with MetS had abnormal ECGs [26]. According to other studies, 61.8% of men and 49.9% of women with MetS had abnormal ECGs [25]. 

Among the patients in our study, 13.9% had multiple abnormal ECGs and 27.4% had just one abnormal ECG. Most of these ECG changes were seen in middle-aged and elderly males. There seems to be a greater association between having multiple ECG abnormalities than having only one abnormality in both genders [25].

Ischemic ECGs were detected in 16.3% of our population (11.1% in men and 5.3% in women). Such ischemic ECGs were reported at a similar frequency to those reported in Iranian and Belgian populations for men. Nevertheless, the prevalence of women in our study was lower than that of Iranians and Belgians [21,27]. Differences in ethnicity and lifestyle could be responsible for the latter difference.

Middle-aged and elderly adults had 11.1% of prolonged QTc cases (1.4% in men and 9.6% in women). Traditionally, QTc and QT have been used in electrocardiography to assess ventricular repolarization [28]. Obese subjects had significantly longer QTc and QT than those with normal weight [28]. Obesity results in longer QT and QTc intervals due to increased sympathetic activity that reduces heart rate variability [22]. MetS patients in our study had a similar prevalence of prolonged QTc to those in other studies [26].

As the most prevalent MetS component in our study, high FBS may represent a potential mechanism affecting ECG. The ECG changes in the FBS group were 9.1% ischemic ECG, 8.7% prolonged QTc, 5.8% AV block (first and second degrees), 4.3% sinus bradycardia, 5.8% BBB, and 0.5% PVCs, AF, ventricular preexcitation, and sinus arrhythmia. Increased FBS levels are linked to hypertension, hyperlipidemia, and prothrombotic states, which all affect electrocardiograms [29]. Atherosclerosis can also increase myocardial ischemia since FBS increases [29].

As the second most prevalent MetS component among our study participants, central obesity represents another potential mechanism for affecting ECG. In the central obesity group, 10.6% of patients had ischemic ECGs; 8.7% had prolonged QTc; 4.8% had first-degree AV block; 2.9% had sinus bradycardia; 5.3% had BBB; 1.4% had PVCs; and 0.5% had PACs. Obesity's effects may be due to the increase in hormone production by adipose tissue, which may result in changes in electrophysiology [30]. In addition, obesity may increase cardiac loading, resulting in remodeling of the heart muscle and, finally, PR prolongation [30].

Limitations

A major limitation of this study was that it was based only on resting ECG findings, and not on clinically symptomatic or angiographically documented CAD. Its low cost, ease of interpretation, and wide availability make ECG a useful tool in studies involving large populations. As a second limitation, our study participants did not represent a random sample of the general population, although they were likely representative of a modernized urban population. Third, our analysis was retrograde, not longitudinal, suggesting a need for additional follow-up study.

## Conclusions

Saudi Arabian populations with MetS were strongly associated with abnormal ECG findings, particularly ischemic ECG findings, AV block (first and second degrees), and BBB (RBBB, LBBB). Middle-aged and elderly males accounted for the majority of these ECG changes. The most important factors contributing to ECG changes were elevated FBS and central obesity.
